# Research Frontier in Chaos Theory and Complex Networks

**DOI:** 10.3390/e20100734

**Published:** 2018-09-25

**Authors:** Guanrong Chen, Marius-F. Danca, Xiaosong Yang, Genaro J. Martinez, Hai Yu

**Affiliations:** 1 Department of Electronic Engineering, City University of Hong Kong, Hong Kong, China; 2 Department of Mathematics and Computer Science, Avram Iancu University, 400380 Cluj-Napoca, Romania; 3 Romanian Institute of Science and Technology, Str. Ciresilor nr. 29, 400487 Cluj-Napoca, Romania; 4 School of Mathematics and Statistics, Huazhong University of Science and Technology, Wuhan 430074, China; 5 Instituto de Ciencias Nucleares, Centro de Ciencias de la Complejidad, Universidad Nacional Autónoma de México, 04510 D.F. Ciudad de México, Mexico; 6 Software College, Northeastern University, Shenyang 110169, China

In recent years, as natural and social sciences are rapidly evolving, classical chaos theory and modern complex networks studies are gradually interacting each other with a great joined development. In particular, the notion of complex networks is becoming a self-contained interdiscipline. Network science as a whole has merged with the basic research and real-world applications of chaos theory, forming one of the most active fields in cognitive science, data science, cloud computing, social sciences, artificial intelligence, and the like.

The theme of this special Issue is on the current research efforts and progress in the promising field of chaos theory as well as complex networks. It comprises 17 selected manuscripts primarily involving four types of subjects, namely theoretical and characteristic analysis of chaotic dynamics, control systems and synchronization, complex networks, and chaos-based applications.

## 1. Theoretical and Characteristic Analysis of Chaotic Dynamics

The paper “Some iterative properties of (F1,F2)-chaos in non-autonomous discrete systems”, by Tang et al. [1] is primarily concerned with invariance (F1,F2)-scrambled sets under iterations. It presents several properties of (F1,F2)-chaos, strong (F1,F2)-chaos and strong F-chaos, and discusses the problems of generically (F1,F2)-chaos, F-sensitivity, etc. Encouragingly, its main results can be considered as an extension of the compound invariance of Li–Yorke chaos and distributional chaos.

Chaotic systems with hyperbolic sine nonlinearity has gained the interest of many researchers. The paper “An approach for the generation of an nth-order chaotic system with hyperbolic sine” by Liu et al. [2] proposes a new approach for generating a class of simple chaotic systems with hyperbolic sine, using two back-to-back diodes to approximate the hyperbolic sine nonlinearity without any multiplier or sub-circuits. The experimental results demonstrate that the physical circuits are very easy to construct by the proposed method, making it possible for them to achieve both physical simplicity and analytic complexity at the same time.

The paper “Lyapunov exponents of a discontinuous 4D hyperchaotic system of integer or fractional order” by Danca [3] proposes a three-dimensional representation of the local finite-time LEs spectrum of a discontinuous dynamical system of integer order or fractional order, which could leads to some comprehensive understanding of the nature.

In the paper “Searching for chaos evidence in eye movement signals”, Harezlak and Kasprowski [4] explore eye movement dynamic features in terms of chaotic dynamics, using nonlinear time series analysis methods. An application to individuals affected by abnormality in the eye functioning, or those suffering from brain diseases, would provide new insights into the usefulness of the presented method.

In the paper “On chaos in the fractional-order discrete-time unified system and Its control synchronization”, Khennaoui et al. [5] introduce a fractional map based on the integer-order unified map and discuss its dynamics and bifurcations. They also propose a one-dimensional adaptive control strategy for forcing the system states towards zero asymptotically, aiming to achieve the complete synchronization of a pair of fractional unified maps with identical or non-identical parameters. Numerical results are presented to confirm the success of the proposed synchronization schemes.

## 2. Control Theory and Synchronization

Compared with real-valued neural networks, synchronization of fractional-order complex-valued neural networks (FOCVNN) has more complicated properties and dynamical behaviors. The paper “Synchronization in fractional-order complex valued neural networks” by Zhang et al. [6], discusses FOCVNN at the presence of a time delay. It designs an error-feedback controller using the comparison theorem of linear fractional-order systems with delay and a fractional inequality, which can be easily applied to achieve synchronization of FOCVNN with delay and improves the existing results.

Based on adaptive control and fractional-order Lyapunov-like function method, Li et al. [7] in their paper “Adaptive synchronization of fractional-order complex-valued neural networks with discrete and distributed delays” studies the information synchronization of drive-response fractional-order complex-valued neural networks (FCVNNs) with discrete and distributed delays. To ensure the states of two FCVNNs with discrete and distributed delays to achieve complete synchronization rapidly, they derive some sufficient conditions using adaptive control technique and Lyapunov function theory. Numerical simulations show the effectiveness of the theoretical results.

In the paper “Output-feedback control for discrete-time spreading models in complex networks”, Alarcón et al. [8] analyzes a Markov chain-based model for Susceptible-Infected-Susceptible (SIS) spreading dynamics over complex networks, and proposes a control mechanism to stabilize the epidemic extinction state. They derive conditions for positioning and identification of actuators and sensors, as well as sufficient conditions for the exponential stability of a desired distribution. A linear feedback control scheme is tested for the stabilization of the extinction state in numerical simulations, showing the good performance of the approach.

In the paper “A novel fractional-order chaotic phase synchronization model for visual selection and shifting” by Lin et al. [9], a two-layer fractional-order chaotic network is constructed, which can simulate the mechanism of visual selection and shifting. The first layer of the model is designed for image segmentation. The second layer, acting as a control function of the cognitive system, implements attention shift by time division phase synchronization between the network and an oscillator. Compared with the typical model of visual information encoding and transmission, the proposed model corresponds better to the human visual system, which opens up new possibilities for further analysis of the mechanism of the human cognitive system.

## 3. Complex Networks

The paper “State estimation for general complex dynamical networks with incompletely measured information” by Wang et al. [10] proposes an approach to reconstructing uncertain state variables of a general complex dynamical network with random and incomplete measurements of transmitted information. Unlike the existing literature, their work is able to balance the excessively deviated estimators and performs well under the influence of incomplete measurements. Especially, there is no special limitation on the node dynamics. To ensure obtaining proper estimator gains with known model parameters, sufficient criteria are derived by employing the Lyapunov stability theory along with the stochastic analysis method. Illustrative examples are given to show the effectiveness of the proposed approach.

By combining the characteristics of network structure with node attributes, Meng et al. [11] propose a novel coupled node similarity (CNS) measure to capture both explicit and implicit interactions between nodes in their paper “Coupled node similarity learning for community detection in attributed networks”. CNS is used to generate the edge weights and then transfer a plain graph to a weighted graph. Furthermore, clustering algorithms are performed on the weighted graphs to detect community structures. By comparing it with the state-of-the-art node similarity measures, experimental results verify the effectiveness of the CNS-based community detection algorithms on several datasets.

Although the classic sampling strategies can provide an unbiased estimate of the population variable, their estimators are not available when the surveys contain sensitive questions. To overcome this limitation, Chen et al. [12] in their paper “Inferring the population mean with second-order information in online social networks” develop an approach to infer the population variable when the respondents’ own characteristics are unknown or not reliable in online surveys. Instead of collecting respondents’ own properties, they use the second-order information of respondents, which is easy to obtain from online social networks. Simulation results show that the proposed approach is able to generate population estimates with high accuracy without knowing the respondents’ own characteristics.

Blakely et al. [13] introduce a new tool for studying the dynamics on complex networks in their paper “Analytic solution for a complex network of chaotic oscillators”, with analytic solutions derived for networks of chaotic hybrid oscillators. In particular, the paper shows a form of coupling between nodes that preserves the solvability of the nodes themselves. Their efforts are focused on applying the model to the study of topics of current interest in network science, such as explosive transitions, symmetry breaking, and control.

In the paper “Quantifying the effects of topology and weight for link prediction in weighted complex networks”, Liu et al. [14] study how to measure various factors in link prediction for weighted networks. They provide a general framework for quantifying the impact of the network topology, weight correlation and statistics on link prediction in weighted networks, by constructing a set of null models. Through experiments on some empirical networks, they found that there is a rich-club phenomenon in the original networks, and that a non-rich club network therein has the strongest randomness. When a network has a strong assortativity of node strengths, the success of network link prediction is relatively high, and is not affected by the change of the weights. Experimental results show that the proposed framework is versatile, which is not only useful for link prediction but also applicable to other aspects of complex networks.

## 4. Chaos-Based Applications

Based on the Logistic map and considering delays, Li et al. [15] propose a new one-dimensional (1D) delay and linearly coupled logistic chaotic maps (DLCL) in their paper “A novel delay linear coupling logistics map model for color image encryption”. They show that this new model has a relatively simple structure, excellent ergodicity property, good sensitivity and better chaotic properties. Through analysis of the algorithm performance, it was found that this algorithm can resist some common attacks, such as brute force attack, differential attack, statistical attack, chosen plain image attack, and noise attack. Therefore, this algorithm has relatively better encryption performance than many other algorithms, and is more effective for image encryption applications.

The paper “2D Tsallis entropy for image segmentation based on modified chaotic bat algorithm” by Ye et al. [16] uses a modified chaotic bat algorithm (MCBA) to develop a 2D Tsallis entropy-based method for gray-level images segmentation. It employs MCBA to look for the best combination of all the parameters. Results of the proposed method are compared with that of other meta-heuristics algorithms, demonstrating that the proposed method has better performances.

The paper “Recommending queries by extracting thematic experiences from complex search tasks” by Zhao et al. [17] investigates the provision of subtask-oriented query recommendations, by extracting thematic experiences from the rich form search logs of complex search tasks logged in a proposed visual data structure. The extracted subtasks are then combined into a network, and query recommendations are provided by applying personalized PageRank on the network. The visual-based subtask identification method and the query recommendation method are evaluated for both informative and tentative search tasks, showing good performances.

In conclusion, the Guest Editors wish to see that this special issue could help to disseminate new information and idea, and to inspire the readers in further exploring the interacting fields of chaos theory and complex networks.
